# Advances in pharmacokinetic-pharmacodynamic modeling for anesthesia, 1987–2024: a review

**DOI:** 10.3389/fphar.2026.1741851

**Published:** 2026-02-02

**Authors:** Yara Tulbah, Ibrahim Aljamaan

**Affiliations:** Biomedical Engineering Department, College of Engineering, Imam Abdulrahman Bin Faisal University, Dammam, Saudi Arabia

**Keywords:** anesthesia control, dose optimization, nonlinear mixed-effects, patient-specific covariates, pharmacodynamic prediction, pharmacokinetic modeling, population variability

## Abstract

In the field of individualized anesthesia, pharmacokinetic-pharmacodynamic (PKPD) models are crucial as they assist in determining the appropriate dosage for various patient groups. This research reviews the development of primary PKPD anesthetic models proposed in the literature from 1987 to 2024. The results from 33 studies are combined, offering a range of concepts from the earliest contributions, such as the “pharmacokinetic mass” concept of Shibutani et al. for fentanyl in obese patients, to the most recent developments in multi-input control strategies for managing anesthesia depth with propofol and remifentanil. Recent advancements are also discussed, including the propofol model proposed by Braathen et al. for severe obesity, which employs innovative scaling techniques to enhance dose accuracy. This study examines physiologically based modeling approaches, reviews traditional compartmental models, and highlights the use of nonlinear mixed-effects modeling. The review concludes by outlining future research aimed at creating more individualized, closed-loop anesthetic delivery systems, emphasizing significant developments in PKPD modeling and identifying limitations in the existing techniques.

## 1 Introduction

General anesthesia is a medical procedure that results in loss of memory, loss of pain perception, relaxation of the muscles, suppression of the autonomic nervous system, and loss of consciousness (Dodds, 1999). Mechanical ventilation can assist with inadequate breathing, but it may also compromise the airway, necessitating the use of a laryngeal mask or an endotracheal tube. Prior to the implementation of enhanced practices and rigorous monitoring, anesthetists faced the risk of overdosing patients. In 1937, Guedel developed a four-stage classification system for anesthetics (Keys, 1975).

During the first stage, known as the induction stage, the patient is sedated but not yet unconscious (Winterberg et al., 2018). The second stage, often referred to as the excited stage or delirium stage and characterized by elevated airway sensitivity and delirium, can pose challenges during airway manipulation (Douglas, 1958). The surgical anesthetic stage, or stage 3, is further divided into four planes to demonstrate the extent of reflex inhibition and muscle relaxation (Hedenstierna and Edmark, 2015). Stage 4, sometimes referred to as the deep stage, represents an overdose that can lead to respiratory failure and circulatory collapse if not treated promptly (Douglas, 1958; Mayer et al., 2018). Safety protocols and ongoing technological advancements have significantly reduced mortality rates associated with anesthesia (Stiegler and Ruskin, 2012; Morriss et al., 2019; Albin and Nikodemski, 2018). However, some argue that more modern methods (Morgans and Graham, 2018) have rendered Guedel’s classification obsolete (Shafiq et al., 2018). Nonetheless, the term is still occasionally used to refer to inhalation induction methods (Bhargava et al., 2004).

Anesthetic medications are administered intravenously (IV) or intramuscularly (IM) to induce drowsiness, provide pain relief, or induce unconsciousness for surgical procedures (Niazi and Matava, 2019). The rapid onset and control over anesthesia depth offered by this analgesic technique are beneficial for stabilizing patients during surgery (Butterworth IV et al., 2013).

Induction medications such as propofol or thiopental are used in conjunction with sedatives or muscle relaxants to enhance patient comfort (Butterworth IV et al., 2013). Continuous monitoring using devices such as blood pressure monitors, Electrocardiograms (ECGs), and pulse oximeters ensure stable vital signs, allowing anesthetists to adjust dosages as needed (City of Hope, 2022). Anesthesia is divided into four stages: induction, maintenance, emergence, and recovery. During induction, intravenous medications such as propofol or etomidate are administered to rapidly induce unconsciousness (Butterworth IV et al., 2013; City of Hope, 2022). Maintenance involves adjusting dosages to maintain an appropriate anesthetic depth under real-time monitoring (Niazi and Matava, 2019; Butterworth IV et al., 2013). Emergence entails progressively lowering anesthetic levels to facilitate a smooth return to consciousness (Butterworth IV et al., 2013; City of Hope, 2022). Finally, recovery in the post-anesthesia care unit involves monitoring for any residual effects to ensure patient safety (Mahoney et al., 2017).

Pharmacokinetics (PK) and pharmacodynamics (PD) are fundamental concepts in many branches of pharmaceutical and medical sciences. In biopharmaceuticals and biologicals for inflammatory bowel diseases, PK studies focus on how medications are metabolized in the body, while PD research examines the effects of treatments. Comparative Pharmacokinetic-pharmacodynamic (PK-PD) studies in healthy individuals are often employed to confirm clinical equivalence and safety, with therapeutic drug monitoring enhancing treatment precision (Chaemsupaphan et al., 2024; Mascarenhas-Melo et al., 2024). Similarly, PK-PD modeling is utilized in toxicology to relate chemical concentrations to biological effects. To improve toxicity predictions, researchers employ pharmacokinetic-pharmacodynamic (PK-PD) and in vitro–in vivo extrapolation models, and artificial intelligence (AI) is being applied to advance research in this field (Kleinstruer and Hartung, 2024; Hoffmann et al., 2017).

For improving health and preventing disease, PK and PD are essential for ensuring the safety and efficacy of macronutrients, micronutrients, and phytochemicals, such as polyphenols and carotenoids, in nutraceuticals (Puri et al., 2022; Faienza et al., 2024). The application of PK-PD concepts optimizes vaccination formulations and administration through the use of adjuvants and virus-like particles to induce effective immune responses (Le et al., 2020; Brisse et al., 2020). By employing PK to tailor treatments at the molecular level, we can use radiopharmaceuticals to minimize adverse effects and enhance efficacy (Dhoundiyal et al., 2024). In cell therapy, gene therapy, and nanomedicine, PK-PD is crucial for maintaining safety and efficacy, particularly in optimizing delivery and therapeutic outcomes (Ay and Reinisch, 2024; Soares et al., 2018).

PK and PD, which influence how medications move through the body and their effects, are critical. The timing of combinations is important because PK interactions affect drug concentrations and can influence distribution, elimination, and absorption. Clinicians utilize PK predictions, prioritize effects, and consider elimination rates and recovery patterns to provide appropriate anesthetics (van den Berg et al., 2017; Roberts and Freshwater-Turner, 2007). This study provides a comprehensive examination of the advancements in anesthetic modeling from 1987 to 2024, focusing on the transition from basic PK models to intricate, patient-specific techniques. By examining how models currently account for factors such as age, weight, genetic variability, and control systems, the study illustrates the trend toward precision dosing in anesthesia.

Readers can expect to discover how these evolving models enhance efficacy, emphasizing the critical role that tailored approaches play in modern anesthetic treatment. The studies reviewed in this paper were selected for their contribution to mathematical PK–PD modeling, with emphasis on compartmental structures and nonlinear mixed-effects (population) frameworks; accordingly, the scope is restricted to 33 clinically relevant PK–PD studies with clearly defined model structure and interpretable parameterization. To reflect major methodological transitions without expanding into parallel model families, two to three representative models were highlighted per decade, and quantitative comparisons are reported only where studies used consistent assessment metrics, summarized explicitly in a “consistent values” table. Methodologically, this review followed a structured workflow of study identification and screening, eligibility-based selection of clinically relevant compartmental/nonlinear mixed-effects PK–PD models, standardized data extraction, and narrative synthesis supported by decade-wise grouping and like-for-like comparison using only consistently reported evaluation metrics. The papers are organized as follows: Section 2 presents a timeline of key advances from 1987 to 2024; Section 3 highlights the most influential models per decade; Section 4 summarizes common model approximations and study inputs/outputs/data sources; Section 5 compares advantages and limitations with respect to predictive performanc; Sections 6–8 provide discussion and conclusions; and Section 9 outlines future work.

## 2 Timeline of aanesthesia modeling

This section provides a timeline of 33 most significant studies from 1987 to 2024. The section describes how anesthesia PK–PD modeling progressed from early two- and three-compartment formulations toward more advanced approaches. While some models explicitly build on prior frameworks (e.g., pediatric extensions, obesity scaling, covariate refinement), others were developed independently from general PK–PD principles rather than derived from a single predecessor. This evolution toward complex, closed-loop control systems employs techniques such nonlinear mixed-effects and physiologically based PK-PD modeling, establishing the context for upcoming discussions on popular model approximations.

### 2.1 Anesthesia modeling between 1987 and 1990

**FIGURE 1:**
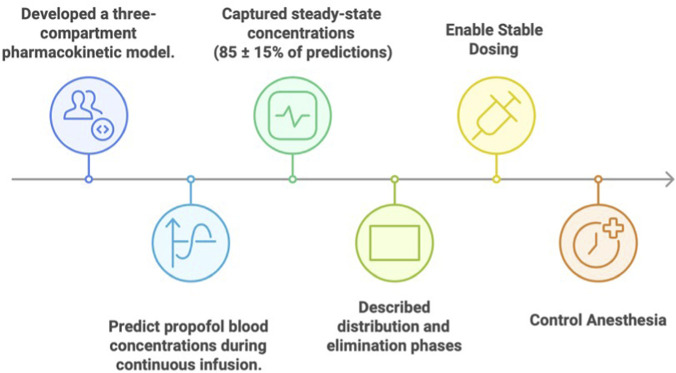
Gepts et al. results (1987).

To forecast the distribution of propofol during infusion, Gepts et al. (Gepts et al., 1987) created a three-compartment model open-loop; validity via fitting measured arterial/whole-blood propofol concentrations under constant-rate infusions in surgical patients; Implementation: not described as a controller/device (modeling/estimation), with central elimination in 1987 (Colburn, 1981; Schwarz, 1978). As demonstrated in (Peck et al., 1984; Schwarz, 1978), using triexponential equations, they fitted blood concentration data and refined the parameters for precise steady-state prediction. By recognizing three distinct distribution phases, this single-input single-output (SISO) model modification of the compartment model previously presented by Westmoreland et al. (Colburn, 1981)-allows for the estimation of concentration at different dosing rates, facilitating effective anesthesia management (Gepts et al., 1987). The prediction outcomes of the model are shown in Figure 1.

### 2.2 1991–1999

**FIGURE 2:**
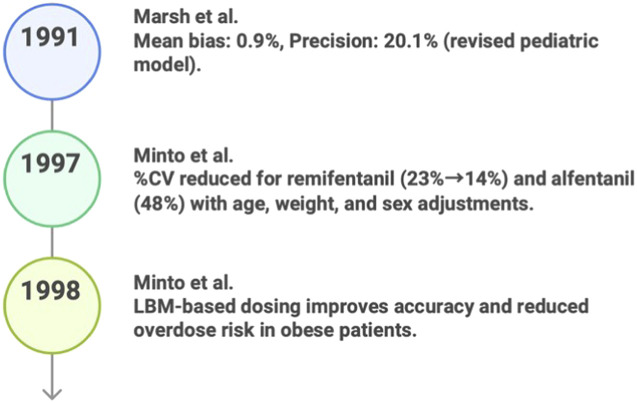
Key predicative studies results in PK/PD (1991–1999).

Between 1991 and 1999, the development of PKPD models for anesthesia steadily progressed toward more accurate and customized dosing approaches. In 1991, Marsh et al. opened-loop (computer-controlled/model-driven infusion; no feedback stated); Validation: pediatric blood-concentration data; Implementation: computer-controlled infusion model with microconstant selection. Developed an innovative method for determining the optimal response to the following problem (Marsh et al., 1991): The modified SISO model improved prediction accuracy by reducing its prediction bias to 0.9% and increasing the forecast accuracy to 20.1%. The central compartment volume and rate constants were adjusted to better fit pediatric physiology, aiming to lower the risk of dosage errors in children (Gepts et al., 1987; White and Kenny, 1990; Browne et al., 1990; Kenny and White, 1990). The expected outcome advancements of the model are shown in Figure 2.

Building on these early adaptations, researchers further refined the model in the mid-1990s. In 1996, Egan et al. (1996) developed a three-compartment PKPD model with Open-loop (no closed-loop control stated); Validation: clinical controlled-anesthesia PK/PD data analyzed in NONMEM + simulation checks; Implementation: NONMEM mixed-effects PK–PD analysis software to compare remifentanil with alfentanil (Shafer and Stanski, 1992). As remifentanil is characterized by a high clearance and a short turnaround time (Glass et al., 1993; Hughes et al., 1992), the model allows for accurate and effective adjustments to anesthesia (Hull et al., 1978). Incorporating multiple inputs (dosages, infusion rates) and outputs (effect-site, blood concentrations) (Holford and Sheiner, 1981; Wagner, 1976; Shafer and Varvel, 1991), the study also demonstrates the possibility of safe, individualized dosing for controlling anesthesia depth.

By 1997 Bailey and Shafer (1991), Minto et al. (1997a) Open-loop; Validation: healthy-adult data with external validation against additional participants; Implementation: GAM → NONMEM covariate-modeling workflow. Had created a remifentanil PKPD model that incorporated age and lean body mass as parameters. Using NONMEM models and EEG data (Bailey and Shafer, 1991; Hughes et al., 1992), they reduced clearance variability (CV) (Shafer and Varvel, 1991) and increased dose prediction accuracy. This SISO framework, derived from previous models (Egan et al., 1996), was designed to promote anesthetic specificity at the individual level and maintain the proper degree of control by titrating bolus dosages and infusion rates for elderly patients. Figure 2 displays the expected outcomes of the model.

Later in 1997, Minto et al. (1997a), Minto et al. (1997b) developed a PKPD model for remifentanil using a three-compartment structure based on pre-existing PKPD models (EEG/effect-site): Not stated as closed-loop (computer-controlled infusion targeting EEG effect, but feedback control not explicitly stated); Validation: EEG-based effect evaluation + simulations across age/LBM; Implementation: computer-controlled infusion algorithm (device not specified) (Kataria et al., 1994; Mandema et al., 1992a). Their use of age and lean body mass covariates to estimate parameters, using the NONMEM and generalized additive models (Sheiner and Beal, 1982; Hastie et al., 1993), produced a better prediction model (Varvel et al., 1992). This SISO paradigm is centered on the objective of PC-based personalized therapy for effective anesthesia. Figure 2 displays the expected results of the model.

In 1998, Egan et al. (1998) open-loop; Validation: obese vs. lean clinical concentration–time data with NONMEM diagnostics; Implementation: NONMEM population PK model to scale key parameters to lean body mass (LBM) when creating a PK model for remifentanil. Built on Egan et al. (1996; remifentanil vs. alfentanil) (Egan et al., 1996) by extending remifentanil PK to obese vs. lean populations and performing covariate testing; the final NONMEM model scaled clearance and distribution volumes to lean body mass (LBM), indicating TBW-based dosing can overpredict concentrations in obesity. By improving dosage prediction accuracy through LBM-based scaling (Egan et al., 1998), our SISO model, which is based on previous opioid models (Egan et al., 1996; Minto et al., 1997b), promotes safe, tailored anesthetic management by reducing the risk of overdose in obese patients.

### 2.3 2000–2009

**FIGURE 3:**
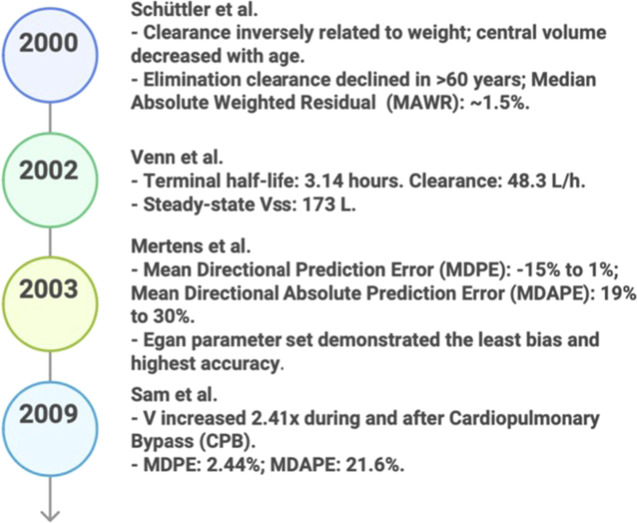
Key predicative studies results in PK/PD (2000–2009).

For more individualized anesthetic dosage, the field of PKPD modeling emerged between 2000 and 2009. Schüttler and Ihmsen (2000) developed a three-compartment PK model for propofol in 2000 and the validation-internal split (development vs. test subsets), based on a two-compartment model described in (Marsh et al., 1991). They assessed the model parameters using a population method in NONMEM. Therefore, by incorporating covariates such as body weight and age, this multi-input, multi-output (MIMO) model was able to predict the parameters accurately, with the drug’s clearance declining with age (Schnider et al., 1998). Figure 3 displays the model’s results.

Using nonlinear mixed-effects modeling, Venn et al. (2002) presented a two-compartment PK model for dexmedetomidine in intensive care unit (ICU) patients in 2002 and the validation-clinical ICU patient data (postoperative) (Venn et al., 1999). This MIMO model, based on the models of Shafer and Varvel and Minto et al., considers patient variables (weight/age) and describes sedation with high clearance, steady distribution, and quick recovery without complications (Venn et al., 1999; Belleville et al., 1992). The findings of the model are shown in Figure 3.

Mertens et al. (2003) introduced three-compartment closed-loop-TCI feedback; validation-MDPE/MDAPE vs. measured concentrations; implemented in a Target-Controlled Infusion (TCI) device, paradigm for remifentanil in 2003. System identification techniques were used to assess the model’s prediction performance (Egan et al., 1996; Minto et al., 1997b). Several parameter sets were evaluated, and the Egan set was shown to be the most effective (Egan et al., 1996). This MIMO model can maintain drug plasma concentrations during anesthesia (Egan et al., 1996) and considers the patient’s characteristics. It is based on previous research by Minto et al. (1997b) and Egan et al. (1996). Figure 3 displays the model’s results.

The developments continued in 2004 when Egan et al. (2004) presented a two-compartment PKPD model for remifentanil, Validation-observed vs. predicted concentrations (with simulations). NONMEM software was used for population analysis, and age was considered for tailored treatment (Schnider et al., 1998). By considering the patient’s age and weight (Schnider et al., 1998), this MIMO technique, based on the work of Minto et al. (1997b), helps to ensure safe remifentanil dosing by simulating sedation and respiratory depression (Egan, 1995).

In 2005, Shibutani et al. (2005) proposed fentanyl dose schedules that incorporated “pharmacokinetic mass” (Shibutani et al., 2004) to increase accuracy in obese individuals. Validation-reported fit statistics. This nonlinear SISO model, with a total body weight (TBW) correction, may yield stable plasma concentrations with a dose accuracy of ±30% (Shibutani et al., 2004), according to Shafer’s work (Shafer et al., 1990). The strategy ensures that patients receive fentanyl at the appropriate dosages and timings for effective analgesia by avoiding accidental overdosing (Shibutani et al., 2004).

Yassen et al. (2006) developed a three-compartment PKPD model and validity is bootstrap model validation in 2006 to explain the pharmacokinetics of buprenorphine and used the NONMEM nonlinear mixed-effects modeling framework to simulate analgesic effects (Mandema et al., 1992a; Jonsson and Karlsson, 1999). The terms keo, kon, and koff, which were previously discussed, refer to the kinetics of opioid receptor association and dissociation, as well as drug absorption and elimination (Yassen et al., 2005; Boas and Villiger, 1985). This MIMO model, which is based on the bio-phase and receptor binding models in (Yassen et al., 2005; Boas and Villiger, 1985; Beal and Sheiner, 1999), enhances personalized pain management by providing accurate and ceiling-free impact predictions.

Peeters et al. (2006) developed a PKPD model for midazolam in nonventilated neonates in 2006 using NONMEM. According to Mulla and Rey’s research, this two-compartment model accounts for factors such as age, weight, and enzyme activity. It effectively simulates sedation through the use of COMFORT-B (Comfort Behavior) ratings and Bi-spectral Index (BIS) monitoring. A variety of inputs ensure that every patient receives a safe and appropriate dosage (Beal and Sheiner, 1999; Blumer, 1998; Stevens et al., 2003; Mandema et al., 1992b; Carnevale and Razack, 2002; Ista et al., 2005; Davidson et al., 2001).

Finally, in 2009, Sam et al. (2009) presented a one-compartment PK model for pediatric remifentanil dosing in cardiac surgery patients using NONMEM and the validation-MDPE/MDAPE reported. built on Minto et al. (1997b) by showing the adult model over-predicted concentrations and then proposing a pediatric model where volume of distribution increases during and after cardiopulmonary bypass (CPB) while clearance remains unchanged, improving fit for infants/children undergoing bypass. The model was adjusted for cardiopulmonary bypass (Davis et al., 1999; Michelsen et al., 2001) and used age and weight inputs to determine plasma concentrations, achieving a median prediction error of 2.44%.

### 2.4 2010–2019

**FIGURE 4:**
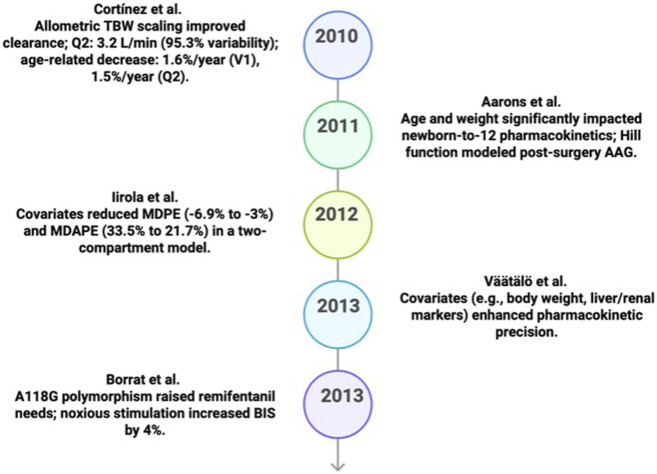
Key predicative studies results in PK/PD (2010–2014).

Between 2010 and 2019, significant changes were made to the PK and PKPD models to enhance tailored anesthetic dosing. In 2010, Cortínez et al. (2010) developed a three-compartment PK model for propofol in obese individuals using NONMEM and allometric scaling based on TBW with validity PC/VPC (predictive checks/visual predictive checks) reported. This MIMO model extends Schnider and Marsh’s models (Schnider et al., 1998; Marsh et al., 1991) by including age as a covariate. It provides precise clearance and distribution estimates, especially for patients who are obese, thereby improving dose precision. Figure 4 shows the expected outcomes of the model.

Additional advancements toward pediatric and critically ill patient models were made in the ensuing years. Using NONMEM with nonlinear mixed-effects modeling, Aarons et al. (2011) developed a two-compartment PK model for ropivacaine and PPX in the pediatric population in 2011 with validation via VPC and bootstrap analysis. Through solid pooled data, our MIMO model improved dosage across age groups by using weight and post-menstrual age as variables for predicting clarity and distribution (Ekström and Gunnarsson, 1996). Figure 4 displays the expected outcomes of the model.

In 2012, Iirola et al. (2012) developed a two-compartment PK model for dexmedetomidine, using NONMEM with nonlinear mixed-effects modeling and the validation via goodness-of-fit diagnostics and prediction-corrected VPC (Venn et al., 2002; Dyck et al., 1993). Using factors such as age, cardiac output, and albumin (Dutta et al., 2000), this MIMO model based on Venn, Lin, and Dyck’s models (Venn et al., 1999; Dyck et al., 1993; Lin et al., 2011) optimized the prolonged ICU sedation dosing in critically ill patients. Additionally, it improved forecast accuracy and decreased error. Figure 4 displays the expected results of the model.

Continuing the trend toward customization, Väätälö et al. (2013) used NONMEM with the SAEM algorithm to construct a population PK model for dexmedetomidine in patients in ICUs in 2013 and evaluation reported using MDPE/MDAPE (prediction-error metrics) on ICU data. This MIMO model accurately predicts clearance and distribution by incorporating factors such as age, body weight, and liver function (Dyck et al., 1993). It improves PK modeling for high-variability populations, but it is not based on any previous model. Figure 4 illustrates the model’s predicted results.

Simultaneously, Borrat et al. (2013) in 2013 devised a PKPD model to investigate the pharmacogenetic influence of A118G polymorphism and model evaluation reported using goodness-of-fit criteria (−2LL/AIC) where LL = log likelihood and AIC = Akaike Information Criterion (Lemmens and Stanski, 2012; Bond et al., 1998; Chou et al., 2006) and noxious stimulation (Lötsch and Geisslinger, 2005; Klepstad et al., 2005; Klepstad et al., 2004) on propofol-remifentanil interactions using NONMEM and a sigmoid Emax model. This MIMO model based on control system principles includes genetic (Lemmens and Stanski, 2012; Bond et al., 1998) and pain factors (Lötsch and Geisslinger, 2005; Klepstad et al., 2005), thus accurately predicting BIS scores for appropriate anesthetic management. Figure 4 presents the model’s predicted results.

**FIGURE 5:**
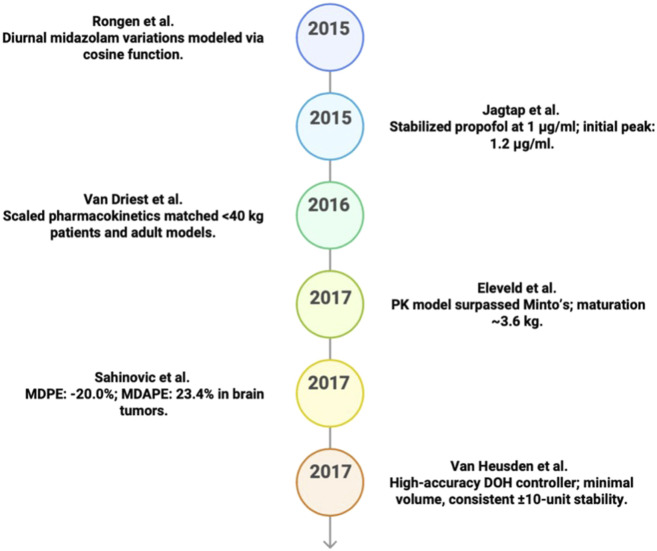
Key predicative studies results in PK/PD (2015–2019).

In 2015, Rongen et al. (2015) developed a three-compartment PK model for midazolam using NONMEM with nonlinear mixed-effects modeling and the validation via bootstrap (250 replicates) and (stratified) VPC (Baraldo, 2008). Incorporating circadian rhythms in bioavailability and clearance with a cosine function (Klotz and Ziegler, 1982), this MIMO model captures 24-hour variations, thereby optimizing dosing accuracy (Klotz and Ziegler, 1982). Built on standard PK principles, it uses time-of-day as an essential input (Baraldo, 2008; Klotz and Ziegler, 1982). Figure 5 shows the model’s outcomes.

Also in 2015, van Rongen et al. (2015) formulated a two-compartment PK model for midazolam in adolescents using NONMEM with nonlinear mixed-effects modeling and the validity of bootstrap and prediction-corrected VPC (Beal et al., 2009). Incorporating body weight as a covariate (Peters et al., 2011; Foster et al., 2012), this MIMO model predicts increased distribution volume with weight while maintaining stable clearance (Brill et al., 2014; Greenblatt et al., 1984). Built on standard PK principles, it accurately predicts drug and metabolite concentrations (Mandema et al., 1992b; Peters et al., 2011).

That same year-2015-Jagtap et al. (2015) developed a three-compartment PK model closed-loop (Proportional-Integral-Derivative (PID)-controlled infusion system); performance reported as reaching 1 μg/mL within 100 s, for propofol using a PID controller to achieve stable dosing. Using system identification, this SISO model maintains target anesthetic levels in blood, muscle, and fat compartments. Independently developed, it rapidly achieves desired concentrations, thus enhancing control over anesthetic depth with patient-specific adjustments (Jagtap et al., 2015). Figure 5 presents the model’s predicted results.

The following year, 2016, Van Driest et al. (2016) constructed a two-compartment PK model for pediatric fentanyl dosing in cardiac surgery using NONMEM and nonlinear mixed-effects modeling with validation via bootstrap analysis (Singleton et al., 1987; Katz and Kelly, 1993; Saarenmaa et al., 2000). Primarily based on body weight, this SISO model accurately predicts plasma levels (Saarenmaa et al., 2000; Foster et al., 2008), thus minimizing invasiveness with remnant samples (Eleveld et al., 2017). Independently developed, it optimizes safe, personalized dosing across pediatric patients (Singleton et al., 1987; Saarenmaa et al., 2000; Foster et al., 2008). Figure 5 displays the model’s predicted results.

In 2017, Eleveld et al. (2017) devised an allometric PKPD model for remifentanil using NONMEM and system identification, scaling parameters model development using covariates (FFM/weight/age/sex); implementation: NONMEM allometric PK–PD model parameterization like clearance and volume (e.g., V1/V2/V3/CL/Q2/Q3, ke0) by fat-free mass (FFM), weight, age, and sex. This MIMO model, based on Minto et al. (1997b), Egan et al. (1998), and La Colla’s models (La Colla et al., 2010), provides accurate predictions across varied populations, thereby enhancing dosing through personalized adjustments. Figure 5 demonstrates the model’s predicted output.

Also in 2017, Sahinovic et al. (2017) developed a PKPD model for propofol in patients with frontal brain tumors using NONMEM and nonlinear mixed-effects modeling, and clinical context reported as target-controlled infusion (TCI); evaluation reported using MDPE/MDAPE (Petersen et al., 2003). Accounting for tumor impact, this MIMO model showed 40% higher clearance in tumor patients, thereby requiring higher infusion rates (Chan et al., 1999). Built on the Schnider et al. (1998), Marsh et al. (1991), and Eleveld et al. (2014) models, it improves dosing precision. The PD component is a sigmoidal Emax model that includes an effect compartment concentration and an explicit “delay parameter” (Delay) to represent the time lag between plasma concentration and BIS response. Figure 5 presents the model’s predicted results.

Finally, van Heusden et al. (2018) in 2017 is closed loop; validated on 80 clinical cases and engineered a MISO control model for anesthesia depth using propofol (stabilizing) and remifentanil (fast-acting) with NeuroSENSE monitoring. Employing system identification techniques and based on the Minto et al. (1997b) and Schüttler and Ihmsen (2000) models, this model maintains the target Depth of Hypnosis (DOH), adapting to patient variability and disturbances, thereby achieving stable and precise control across clinical cases. Figure 5 illustrates the predicted model’s results.

### 2.5 2020–2024

**FIGURE 6:**
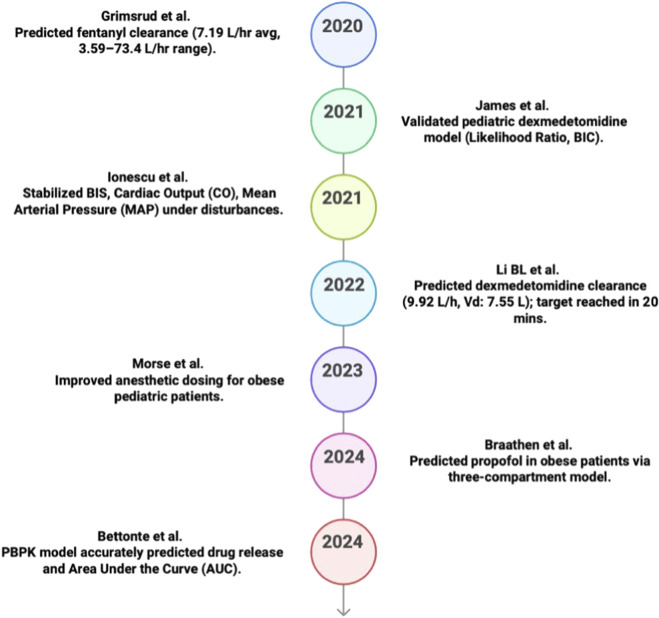
Key predicative studies results in PK/PD (2020–2024).

To Improvements in PK-PD modeling and physiologically based pharmacokinetic (PKPD) modeling between 2020 and 2024 allowed for more individualized dosing plans for specific patient populations. In 2020, Grimsrud et al. (2020) developed a two-compartment PK model for fentanyl in burned children, using NONMEM software and allometric scaling; assessed using AIC/BIC (BIC is Bayesian information criterion) (Choi et al., 2016). Body weight and total body surface area are considered for clearance and distribution to improve the model that represents individual differences (Grimsrud et al., 2018). This MIMO model was developed specifically for burn patients and aids in dose calculation with numerous factors (Reilly et al., 1985). Figure 6 shows the outcomes of the model.

Using NONMEM software, James et al. (2022) expanded on this precise method in 202 by creating a pediatric dexmedetomidine population PK model two-compartment PK model for pediatric dexmedetomidine. To increase the accuracy of the modeling, the model uses weight and post-menstrual age to predict the distribution and clearance, respectively (Kohli et al., 2012). The model can precisely estimate the individual dose requirements of each patient in the ICU based on a wide range of input criteria. Its foundation is an established PK framework. Figure 6 displays the expected results of the model.

Ionescu et al. (2021) also presented a Model Predictive Control (MPC)-based MIMO simulation model for anesthesia and hemodynamic control utilizing MATLAB/Simulink for multi-drug anesthesia modeling/control evaluation in 2021. The model employs five pharmacological inputs and a number of physiological outputs, such as arterial pressure, cardiac output, and degree of anesthesia (Ionescu et al., 2008). It was created independently and addresses patient differences and disruptions to enhance the multiple drug therapeutic control system (Ionescu et al., 2008; Padula et al., 2016. The model’s expected outcomes are shown in Figure 6.

Using NONMEM with allometric scaling and age-based maturation, Li et al. (2022) developed a two-compartment PK model for intranasal dexmedetomidine in the pediatric population for the first time in 2022 and assessed using bootstrap resampling (1,000 datasets) and prediction-corrected VPC. The model employs post-menstrual age and body weight as covariates and is based on the Potts et al. (2008) model. The Monte Carlo analysis validated the precision of its dosage predictions and PK variability in children. The model’s expected outcomes are shown in Figure 6.

A PKPD model was created in 2023 by Morse et al. (2023) to optimize anesthetic dosage in pediatric obesity PK–PD dosing framework providing dosing equations, while accounting for body composition, age, and weight. Based on variables such as fat-free mass, the model precisely forecasts the drug concentration in the patient’s system during anesthesia, ensuring a safe and effective procedure. This approach reduces the risk of pediatric dose errors by addressing the pharmacokinetics linked to obesity. Figure 6 displays the expected outcomes of the model.

To tailor propofol dosage for obese patients, Braathen et al. (2024) proposed a three-compartment PK model using NONMEM software and assessed using prediction-error metrics and bootstrap (allometric scaling exponent 0.75 reported). With a median absolute error of 1.4% and a relatively low bias, the model was created using TBW, LBW, Predicted Normal Weight (PNWT), and allometric scaling with an exponent of 0.75. By incorporating factors related to obesity and an extended sample, it enhances earlier models. The model’s expected outcomes are shown in Figure 6.

To better comprehend drug release and distribution with drug and tissue properties including solubility, viscosity, and inflammation, Bettonte et al. (2024) built a PBPK model for long-acting antiretroviral medications administered via intramuscular injection in 2024. Effective dosage optimization for prolonged therapy is supported by the fact that 83% of the anticipated values were within 1.5 times the observed values when compared to clinical data. The model’s expected outcomes are shown in Figure 6.

## 3 Decadal highlights: key studies transforming PKPD models

This section provides an overview of the development of anesthetic modeling from 1987 to 2024, summarizing foundational contributions. It traces the evolution from the initial three-compartment model by Gepts et al. to complex multi-input control schemes for tailored dosing. It explains how significant research has included pediatric modifications, covariate adjustments (such as age and LBM), and new scaling approaches to increase prediction accuracy. This overview, which selects two to three pivotal studies from each decade, sets the stage for the detailed subsections that follow.

### 3.1 1987: foundational PK model for continuous infusion

#### 3.1.1 Gepts et al. model

In Gepts et al. (1987), a three-compartment PKPD model describes propofol concentrations, using the triexponential Equation 1:

$$∁\left(t\right)={Ae}^{-\propto t}+Be^{-\beta t}+∁e^{-\gamma t}$$ (1)

Where:

- C(t) is the propofol blood concentration at time t;
- A, B, and C are the coefficients of the three exponential terms;
- α, β, and γ are the rate constants of the three exponential terms, representing the distribution and elimination of the drug.

For infusion Equation 2:

$$∁\left(T+t\right)={∁}_{1}\left(1-e^{-\alpha T}\right)e^{-\alpha T}+{∁}_{2}\left(1-e^{-\beta T}\right)e^{-\beta T}+$$

$${∁}_{3}\left(1-e^{-\gamma T}\right)e^{-\gamma T}$$ (2)

Defined as:

- C(T + t) is the propofol blood concentration at time T + t;
- T is the duration of the infusion, and t is the post-infusion time;
- C_1_, C_2_, and C_3_ are the interceptors for each exponential phase;
- α, β, and γ are the slopes (rate constants) of each exponential phase.

### 3.2 1990s: landmark developments

#### 3.2.1 Marsh et al. model (1991-pediatric model)

The pediatric PK model developed by Marsh et al. (1991) built on White and Kenny (1990) and PK constants from Gepts et al. (1987) by retaining the model-driven infusion framework but re-estimating the propofol micro constants for children and increasing the allowable target concentration (15 → 20 μg/mL). Resulted in a major shift in enhancing anesthesia by applying adult-based parameters to children. The three-compartment model provided a better way of describing the kinetics of propofol during an infusion, thereby greatly enhancing predictability while reducing errors (Marsh et al., 1991). Some important enhancements included increasing Vc to mL/kg and modifying the rate constants as in Equation 3:

$$k_{10}=0.1\min^{-1},k_{12}=0.085\min^{-1},k_{21}=0.021\min^{-1},$$

$$k_{13}=0.033\min^{-1},k_{31}=0.0033\min^{-1}.$$ (3)

Where:

- k_10_ is the elimination rate constant;
- k_12_ is the rate constant for transfer to the peripheral compartment;
- k_21_ is the rate constant for transfer from the peripheral compartment;
- K_13_ is the rate constant for the slow peripheral compartment.

These changes, as provided in Equation 3, helped explain the faster distribution and clearance in children and provided a safer standard for pediatric anesthesia.

#### 3.2.2 Minto et al. model (1997-age and lean body mass)

In their study, Minto et al. (1997a) included age and LBM as covariates in a PKPD model for remifentanil, thereby improving dosing accuracy across various age groups. The model incorporated a three-compartment PK structure and an effect-site PD model for individualized anesthesia care. To calculate the volume of distribution at the time of peak effect-site concentration, we use the Equation 4:

$$V_{d,pe=\frac{Bolusdose}{Initialbloodconcentration\times \left(1-Percentdecrease\right)}}$$ (4)

Terms explained:

- Bolus dose: Plain intravenous bolus dose given to the patient;
- Initial blood concentration: Concentration in the blood at time zero;
- Percent decrease: The reduction in blood concentration from time zero to the time of peak effect-site concentration (t peak_k_).

Also, Equation 5 is given as follows:

$$Bolusdose=V_{d,pe}\cdot {E∁}_{50}\cdot \left(1-e^{k_{e0}t_{peak}}\right)$$ (5)

Where:

- V_d, pe_ is the volume of distribution at the time of peak effect-site concentration;
- EC_50_ is the effect-site concentration that produce half of the maximum EEG response;
- k_e0_ is the rate constant representing the turnover between plasma and the effect site;
- t_peak_ is the time required to achieve peak effect-site concentration.

#### 3.2.3 Minto et al. model (1997-EEG and effect-site model)

Minto et al. (1997b) developed a three-compartment PK model and PD framework for remifentanil, integrating EEG data and the Hill equation. The model linked drug concentrations to effects, incorporating age and LBM to improve dosing precision.

The parameter values were remodeled as Equation 6:

$$P_{i}=\theta_{TV}\cdot \exp\left(\eta \right)$$ (6)

Where:

- P_i_ is the value of the parameter in the individual;
- θ^TV^ is the typical value of the parameter in the population;
- η is a rando; m variable with mean zero and variance ω2.

The PD model used the Hill Equation 7:

$$Effect=E_{0}+\left(E_{\max}-E_{0}\right)\cdot \frac{{∁}_{e}^{\gamma }}{{∁}_{e}^{\gamma }+{E∁}_{50}^{\gamma }}$$ (7)

Explained as follows:

- Effect: The observed PD response;
- E_0_: The baseline effect;
- E_max_: The maximum effect;
- C_e_: The effect-site concentration;
- EC_50_: The effect-site concentration producing 50% of Emax;
- γ: The hill coefficient.

### 3.3 2000–2009: advances in personalized anesthesia models

#### 3.3.1 Venn et al. model (2002-pharmacokinetic modeling of dexmedetomidine in ICU patients)

A PK model for dexmedetomidine in ICU patients was developed based on age, weight, and cardiac output. The model, a two-compartment open model with clearance and volume parameters, establishes optimal sedation in critical care (Venn et al., 2002). The following Equation 8 is used:

$$MRT=\frac{AUMC}{AUC}-\frac{AUMCR}{AUCR}$$ (8)

Definitions:

- MRT is the mean residence time—the average time a drug molecule remains in the body;
- AUMC is the area under the first moment curve;
- AUC is the area under the curve.

Also, for clearance, Equation 9 is given as follows:

$$Clearance=\frac{Dose}{{AUC}_{\infty }}$$ (9)

Terms explained:

- Clearance is the elimination rate of the drug;
- Dose is the amount of drug administered;
-
${\text{AUC}}_{\infty }$ is the area under the curve extrapolated to infinity.

And for the volume of distribution at steady state, Equation 10 is as follows:

$$V_{ss}=\frac{CL\times MRT}{1-R}$$ (10)

Explanation:

- V_ss_ is the apparent volume of distribution at steady state;
- CL is the clearance;
- MRT is the mean residence time.

For individual parameter value, Equation 11 is as follows:

$$P_{j}=P_{TV}\times \exp\left(\eta \right)$$ (11)

Where:

- P_j_ is the individual parameter value;
- P_TV_ is the typical (or median) value of the parameter;
- Exp(n) represents the individual variability around the typical value.

#### 3.3.2 Shibutani et al. model (2005-pharmacokinetic mass for fentanyl dosing)

Shibutani et al. (2005) introduced the concept of “pharmacokinetic mass” as a novel metric to refine fentanyl dosing, thereby addressing overdose risks in obese patients. The model equations describe dosing relationships Equations 12–14:

$$Dose\left(\mu g/h\right)=-167\times e^{-\beta \times TBW}+149$$ (12)

This Equation 12 describes the nonlinear relation between dose and TBW; r2 = 0.551, P < 0.001).

And for the linear relationship Equation 13 with pharmacokinetic mass: r = 0.741, P < 0.001,

$$Dose\left(\mu g/h\right)=1.22\times pharmacokineticmass-7.5$$ (13)

For simplified linear relation Equation 14 with minimal deviation (+0.7%) from previous,

$$Dose\left(\mu g/h\right)=1.12\times pharmacokineticmass$$ (14)

Key variables:

- Dose (g/h): Required fentanyl dose per hour, calculated using body weight or pharmacokinetic mass;
- TBW: Total body weight (kg);
- Pharmacokinetic mass: A nonlinear TBW function reflecting fentanyl clearance;
- β: Coefficient in the TBW-dose relation.

Pharmacokinetic mass effectively standardized dosing.

### 3.4 2010–2019: advancing tailored models in anesthesia: pharmacokinetics, dynamics, and control

#### 3.4.1 Cortínez et al. model (2010-allometric scaling for obesity in propofol PK)

Cortínez et al. (2010) built upon Schnider et al. (1998), Marsh et al. (1991) by developing an obesity-capable propofol PK model, introducing allometric scaling with total body weight for clearances/volumes and retaining an age effect on inter-compartmental distribution parameters (Q2, V2) to improve predictions in obese subjects. Utilized the concept of “pharmacokinetic mass” to more accurately determine fentanyl doses for obese patients. The dosing Equation 15 of Cortinez et al. are as follows:

$$P_{i}=P_{TV}\times {\left(\frac{TBW}{70}\right)}^{\theta }\times e^{\eta }$$ (15)

Parameters defined:

- P_i_: Parameter value (e.g., CL and V) for the individual;
- P_TV_: Typical parameter value for the population;
- TBW: Total body weight (kg);
- θn: Allometric exponent;
- e: Variability term (mean = 0, variance = ω2).

And for scaled parameter value Equation 16:

$$P_{i}=P_{std}\times {\left(\frac{X_{i}}{X_{std}}\right)}^{PWR}$$ (16)

Where:

- P_i_: Scaled parameter value;
- P_std_: Standard parameter value;
- X_i_: Individual measure of size;
- X_std_: Standard measure of size;
- PWR: Scaling exponent. For central clearance Equation 17:

$${CL}_{1}={CL}_{1,std}\times {\left(\frac{TBW}{70}\right)}^{0.75}$$ (17)

Represented as follows:

- CL_1_: Central clearance;
- CL_1, std_: Standard clearance value;
- 0.75: Allometric exponent for clearance. And finally, for Peripheral volume Equation 18:

$$V_{2}=V_{2,std}\times e^{{-k}_{age}\times age}$$ (18)

Where:

- V_2_: Peripheral volume;
- V_2, std_: Standard peripheral volume;
- k_age_: Rate constant for the age effect on V_2_.

Key Parameters:

- V_1_: Central volume (4.47L/70 kg);
- V_2_: Peripheral volume (26.6L/70kg, decreases with age);
- V_3_: Peripheral volume (53.8L/70 kg);
- CL_1_: Central clearance (2.25L/min/70 kg);
- Q_2_: Inter-compartment clearance (3.20 L/min/70 kg, decreases with age);
- Q3: Inter-compartment clearance (0.52 L/min/70 kg).

This model effectively standardized propofol dosing by accounting for size and age variations.

#### 3.4.2 Van Heusden et al. model (2017-MISO control for Depth of Hypnosis)

This study (van Heusden et al., 2018) presented a novel MISO system for anesthesia control using propofol and remifentanil with a PID controller and Neuro SENSE monitoring. The design is characterized by the following Equation 19:

The nominal PKPD model is defined as follows:

$$G_{0,PKPD\_R}=\begin{array}{c} \mathrm{argmin}\\ GPKPD\_R \end{array}\sum_{\omega }\begin{array}{c} \max\\ i\epsilon \left[1,N\right] \end{array}\left|M_{Ri}\left(j\omega \right)\right.\left.-GPKPD\_R\left(j\omega \right)\right|$$ (19)

The structure of the reduced controller is Equation 20:

$$K_{red}\left(q^{-1}\right){=K_{fix}\left(q^{-1}\right)b}_{0}+b_{1}q^{-1}+\frac{b_{2}q^{-2}}{1+a_{1}q^{-1}+a_{2}q^{-2}}$$ (20)

The design objective is Equation 21:

$$M_{d}=\frac{G_{0,PKPD\_R}K_{R}G_{NS}}{1+G_{NS}PIDG_{0,P}}=G_{0,PKPD\_R}K_{R}S_{P}$$ (21)

The nonlinear stability constraint is expressed as Equation 22:

$$\left|G_{NS}G_{0,P}\left(j\omega \right)PID\left(j\omega \right)\omega_{I,P}\left(j\omega \right)\right|+\left|G_{NS}G_{0,R}\left(j\omega \right)K_{R}\left(j\omega \right)\omega_{I,R}\left(j\omega \right)\right|\leq$$

$$\left|1+G_{NS}\left(j\omega \right)\left(G_{0,P}\left(j\omega \right)PID\left(j\omega \right)+G_{0,R}\left(j\omega \right)K_{R}\left(j\omega \right)\right)\right|$$ (22)

Explained as follows:

- G_0, PKPD_R_: Baseline PKPD model parameter for remifentanil;
- G_PKPD_R_: Subject-specific PKPD model for remifentanil;
- M_Ri_: A set of N PKPD models;
- G_0,P_: Nominal model for the propofol effect;
- PID: The propofol controller;
- G_NS_: Monitor dynamics;
- G_0,R_: Baseline PKPD model for remifentanil;
- K_R_: Remifentanil controller;
- w_I,P_ and w_I,R_: Uncertainty bounds for propofol and remifentanil, respectively;
- S_P_: The sensitivity function for the propofol controller.

### 3.5 2020–2024: innovations in PKPD modeling for personalized drug delivery and anesthesia management

#### 3.5.1 Ionescu et al. model (2021-multi-Drug simulation for anesthesia and hemodynamics)

This study (Ionescu et al., 2021) presented a MATLAB/Simulink-based simulation model for optimizing anesthetic and hemodynamic management using multiple drug dosage regimens. Employing advanced MPC techniques (including EPSAC), the model incorporates the three-compartment PK model Equation 23:

$$x^{˙}1\left(t\right)=-\left(k_{10}+k_{12}+k_{13}\right)x_{1}\left(t\right)+k_{21}x_{2}\left(t\right)+k_{31}x_{3}\left(t\right),$$

$$\dot{x}2\left(t\right)=k_{12}x_{1}\left(t\right)+k_{21}x_{2}\left(t\right),\dot{x3}\left(t\right)=k_{13}x_{1}\left(t\right)+k_{31}x_{3}\left(t\right).$$ (23)

Specified as follows:

- x_1_(t), x_2_(t), x_3_(t): Drug concentrations in fast and slow compartments (mg/mL);
- k_ij_: Transfer rates between compartments (1/min).

The dose-effect response: A nonlinear Hill Equation 24:

$$E=E_{0}-E_{\max}\cdot \frac{x_{e}^{\gamma }}{{∁}_{50}^{\gamma }+x_{e}^{\gamma }}$$ (24)

Where:

- E(%): Predicted effect of the drug;
- x_e_: Effect site concentration;
- C_50_: Concentrationneededtoobtain50% of the maximum effect;
- γ: Shape parameter.

The Atracurium PK model Equation 25:

$${\dot{x}}_{1}=-\lambda_{1}x_{1}\left(t\right)+a_{1}u\left(t\right),$$

$${\dot{x}}_{2}=-\lambda_{2}x_{2}\left(t\right)+a_{2}u\left(t\right),$$

$${∁}_{p}=x_{1}\left(t\right)+x_{2}\left(t\right).$$ (25)

Specified as:

- x_i_(t)(i = 1, 2): State variables;
- a_1_,a_2_: Parameters(kg/mL);
- λ_1_,λ_2_: Elimination rates (kg/min);
- u(t): Drug infusion rate (µg/kg/min);
- Cp: Plasma concentration (µg/mL).

The Atracurium PD model Equation 26:

$$∁=-\lambda_{∁}∁\left(t\right)+{∁}_{p}\left(t\right),$$

$${\dot{x}}_{e}=-\frac{1}{\tau }x_{e}\left(t\right)+\frac{1}{\tau }∁\left(t\right),$$

$$NMB=100\cdot \frac{{∁}^{\gamma }}{{∁}_{50}^{\gamma }+x_{e}\left(t\right)\gamma }.$$ (26)

Defined as:

- C(t): Intermediate variable;
- x_e_(t): Drug concentration in the effect compartment;
- NMB (%): Neuromuscular blockade level;
- λ,τ(min), C_50_(µg/mL), and γ: Patient-independent parameters.

Effect site concentration Equation 27:

$${\dot{x}}_{e}\left(t\right)={-k}_{e0}x_{e}\left(t\right)+k_{1e}x_{1}\left(t\right)$$ (27)

Where:

- k_e0_: Metabolic rate of the drug;
- k_1e_: Transfer rate from the central compartment (x_1_) to the effect-site compartment (x_e_).

Drug infusion input Equation 28:

$$\frac{u\left(t\right)}{V_{1}}$$ (28)

Described as follows:

- u(t): Drug infusion rate (mg/ml/min);
- V_1_: Volume of the first (blood) compartment.

Matrix form of the model Equation 29:

$$\left[\begin{array}{c} {\dot{x}}_{1}\left(t\right)\\ \begin{array}{c} {\dot{x}}_{2}\left(t\right)\\ {\dot{x}}_{3}\left(t\right)\\ {\dot{x}}_{e}\left(t\right) \end{array} \end{array}\right]=\left[\begin{array}{ccc} -\left(k_{10}+k_{12}+k_{13}\right) & k_{21} & \begin{array}{cc} k_{31} & 0 \end{array}\\ k_{12} & {-k}_{21} & \begin{array}{cc} 0 & 0 \end{array}\\ \begin{array}{c} k_{13}\\ k_{1e} \end{array} & \begin{array}{c} 0\\ 0 \end{array} & \begin{array}{cc} \begin{array}{c} {-k}_{31}\\ 0 \end{array} & \begin{array}{c} 0\\ {-k}_{e0} \end{array} \end{array} \end{array}\right]\left[\begin{array}{c} x_{1\left(t\right)}\\ x_{2\left(t\right)}\\ \begin{array}{c} x_{3\left(t\right)}\\ x_{e\left(t\right)} \end{array} \end{array}\right]+\left[\begin{array}{c} 1\\ 0\\ \begin{array}{c} 0\\ 0 \end{array} \end{array}\right]u\left(t\right)$$ (29)

This comprehensive model offers a robust foundation for simulating drug behavior and optimizing multi-drug dosing strategies.

#### 3.5.2 Braathen et al. model (2024-tailored propofol pharmacokinetics in obesity)

This study (Braathen et al., 2024) developed a three-compartment propofol PK model for very obese patients using allometric scaling and body composition variables (TBW, LBW, and PNWT). For example, the model uses equations such as Equations 30–33, 35:

$$\text{Predicted Normal Weight }\left(\text{PNW}\right)\text{ for males}=1.57\cdot TBW-0.0183\cdot BMI\cdot TBW-10.5$$ (30)

$$\text{LBW for females}=\text{Height }\left(\text{cm}\right)-152.4\;\cdot \;0.9055+45.5$$ (31)

$$\text{LBW for males}=\text{Height }\left(\text{cm}\right)-152.4\;\cdot \;0.9055+50$$ (32)

$$\text{LBW }\left(\text{Lemmens}\right)=22\;\cdot \text{ Height}$$ (33)

$$\text{LBW }\left(\text{Brocca}\right)\text{ for males}=\text{Height}-100$$ (34)

$$\text{LBW }\left(\text{Brocca}\right)\text{ for females}=\text{Height}-105$$ (35)

These Equations 30–34, illustrate how patient-specific variables are used to tailor propofol dosages for obese patients.

## 4 Common empirical approximated models

The tables below are from the Decadal Highlights section. First, Table 1 explains the prediction methods of these studies.

**TABLE 1:** Model name and prediction method for common models.

Model name	Prediction method
Gepts et al. (1987)	System identification, three-compartment model prediction
Marsh et al. (1991)	System identification, PK modeling prediction
Minto et al. (1997a)	NONMEM-based PKPD Modeling
Minto et al. (1997b)	PKPD modeling with NONMEM, machine learning, Generalized Additive Modeling (GAM)
Venn et al. (2002)	Two-compartment model & nonlinear mixed-effects modeling
Shibutani et al. (2005)	Model-based, non-linear adjustment, pharmacokinetic Mass calculation
Cortínez et al. (2010)	Allometric scaling, visual predictive checks, and bootstrapping
van Heusden et al. (2018)	System Identification
Ionescu et al. (2021)	System identification via MPC, utilizing EPSAC and quasi-infinite horizon MPC.
Braathen et al. (2024)	System identification, nonlinear mixed effects modeling via NONMEM.

Second, Tables 2, 3 detail the models’ inputs, outputs, data sizes, and sources, providing insights into the contributions of the models to personalized anesthesia management.

**TABLE 2:** Model name, inputs, outputs, data size, and source (Part 1).

Model name	Inputs	Outputs	Data size and source
Gepts et al. (1987)	Infusion rate	Css, Vc, Cl, Vd, Vss, compartmental Micro constants	18 patients
Marsh et al. (1991)	Patient weight, target plasma concentration, infusion rate	Predicted blood propofol concentration	Data from 18pediatric patients
Minto et al. (1997a)	Age, lean body mass (LBM), remifentanil concentration	Age and LBM affect remifentanil’s EEG impact. EEG correlates with respiratory depression and pain relief. EEG-based dosing adjusts remifentanil by age	Three simulated populations (n = 500 each), based on typical values forages 20, 50, and 80years, with 55 kg LBM.
Minto et al. (1997b)	Age, gender, remifentanil concentration, patient demographic data	Drug effect (sedation levels, BIS), parameter estimates, model predictions	65 healthy adults aged 20–85 years

**TABLE 3:** Model name, inputs, outputs, data size, and source (Part 2).

Model name	Inputs	Outputs	Data size and source
Venn et al. (2002)	Plasma concentrations, infusion rates, blood samples, clinical observations	Pharmacokinetic parameters like half-life, clearance, volume of distribution, Cmax, Tmax, AUC, and MRT, which help describe drug Absorption, distribution, and elimination	From post operative ICU patients, 10patients were monitored for the first 6 h, and five patients were monitored for the remaining 7–14 h
Shibutani et al. (2005)	Total Body Weight (TBW)	Fentanyl Dose	69patients with various body types and abdominal surgery cases
Cortínez et al. (2010)	Propofol dose, Total Body Weight (TBW), Age	Propofol plasma concentration, Clearance rates	51 patients. Open TCI Database, HPLC samples from a clinical study
van Heusden et al. (2018)	Propofol and Remifentanil infusions	Depth of Hypnosis (DOH), Remifentanil Effect Site Concentration (Ce)	138 patients over two phases from clinical study data
Ionescu et al. (2021)	Dosing rates for five drugs (propofol, remifentanil, rocuronium, dopamine/dobutamine, sodium nitroprusside), along with patient-specific inputs (age, height, weight, BIS) and pharmacokinetic parameters (γ, C50)	BIS (hypnosis), RASS (analgesia), NMB (neuro muscular blockade), CO (cardiac output), and MAP (mean arterial pressure)	24 representative patients, simulated data based on patient characteristics
Braathen et al. (2024)	Total body weight, lean body weight, predicted normal weight, sex	Clearance and volume of distribution parameters with a 1.4%median prediction error and a 21.7%median absolute error, predicting propofol concentrations overtime, compared against the Marsh, Schnider, and Eleveld models	474 propofol serum measurements from 69 patients (ages 19–60, BMI 21.6–67.3 kg/m^2^)

## 5 Significant anesthesia prediction model studies

This section compares and contrasts the major anesthesia prediction models mentioned in the Decadal Highlights section. The advantages and limitations of each model are examined, and these comparisons are summarized in Tables 4, 5.

**TABLE 4:** Advantages and limitations of significant anesthesia prediction models (Part 1).

Model name	Advantages	Limitations
Gepts et al. (1987)	• Estimates pharmacokinetic parameters such as Css, Vd, Vss, Vc, and Cl • Enables precise dosage predictions • Maintains a constant and safe drug dose in the body	• No incorporation of adaptive machine learning • Small sample size and limited demographic factors • Potential impact on external validity
Marsh et al. (1991)	• Enhanced pediatric weight-based dosing • Reduced bias from −18.5% to 0.9% • Safe anesthetic administration	• Small sample size (18pediatric patients) • No machine learning or advanced variability recognition • Limited external applicability
Glass et al. (1993)	• Incorporation of age and LBM for individualized dosing • Utilization of EEG data for opioid effect modeling	• High computational demands • Limited generalize ability and rapid dosing instability
Minto et al. (1997b)	• Comprehensive covariate analysis performed using NONMEM. • Improved individualized dosing and prediction accuracy	• Complex model increased bias • Small sample size and single-drug interaction focus
Venn et al. (2002)	• Provision of stable sedation with rapid recovery • Predictable pharmacokinetics for effective critical care sedation	• Advanced modeling requires expertise • Results are inconsistent across applications
Shibutani et al. (2005)	• Weight-based dosing prevents overdose in obese patients • Simple clinical application	• Based solely on total body weight (TBW) • Moderate sample size (69patients)

**TABLE 5:** Advantages and limitations of significant anesthesia prediction models (Part 2).

Model name	Advantages	Limitations
Cortínez et al. (2010)	• Effective for obese patients using allometric scaling • Accurate predictions with small sample datasets	• No incorporation of machine learning • Clinical complexity in application
van Heusden et al. (2018)	• Robust to patient variability and disturbances • Guarantee clinical flexibility for anesthesia depth control	• Possible overshooting during induction or air way manipulation phases • Need for further development for reliability
Ionescu et al. (2021)	• Open-source model allowing flexibility for user modification • Realistic simulations incorporating disturbances	• No direct assessment of pain delivery accuracy • Computational challenges in balancing hemodynamics
Braathen et al. (2024)	• Suitable for severely obese patients • Safe and effective dosing with minimal errors	• Small dataset with limited diversity • Lack of comprehensive sampling for pharmacodynamic modeling

## 6 Consistent prediction-error evaluation metrics reported across studies (MDPE/MDAPE)

Table 6 summarizes the subset of included studies that report prediction performance using the same, directly comparable assessment-error metrics: median prediction error (MDPE) for bias and median absolute prediction error (MDAPE) for inaccuracy. The values are extracted as presented in the reviewed studies to enable a consistent cross-study comparison of model predictive performance.

**TABLE 6:** Consistent prediction-error evaluation metrics reported across studies (MDPE/MDAPE).

Year	Study	MDPE/MDAPE
2003	Mertens et al. (2003)	MDPE ≈ −15% to +1%; MDAPE ≈19%–30% (reported across different target concentration ranges)
2009	Sam et al. (2009)	MDPE = 2.44%; MDAPE = 21.6%
2012	Iirola et al. (2012)	MDPE = −5.9 to −3.7; MDAPE = 33.5 to 21.7
2017	Sahinovic et al. (2017)	MdPE = −20.0%; MdAPE = 23.4% (reported for the primary model in the brain-tumor group)

## 7 Discussion

Among the 33 included studies, delayed drug effect is addressed using the conventional effect-site/link (biophase) formulation (and, in Sahinovic et al. (2017) an explicit delay term), while no fractional-order PK–PD models were identified, consistent with the review’s focuses on clinically established compartmental and mixed-effects frameworks. Clinically, the Marsh et al. (1991) propofol model has been implemented in TCI devices for propofol dosing, and Mertens et al. (2003) evaluated remifentanil delivery under target-controlled infusion (TCI) during anesthesia. In the broader TCI context, this emphasis on effect-site/link formulations is consistent with how TCI systems operationalize “plasma mode” versus “effect-site mode,” where an effect-site compartment is introduced specifically to account for plasma–brain equilibration hysteresis and is parameterized by the equilibration rate constant ke0; this linkage underpins practical dose targeting when direct effect-site concentrations are not measurable and must be inferred via clinical effects (Bidkar et al., 2023).

From a model development and evaluation standpoint, the reviewed body of work reflects the prevailing population PK/PD workflow in which models are typically built for descriptive/interpretive purposes using nonlinear mixed-effects methods (commonly NONMEM), with covariate exploration dominated by demographic and organ-function predictors, and with substantial variability in how model-building steps and validation practices are reported (Dartois et al., 2007). This matters clinically because translation into decision-support (including TCI parameterization and “effect-site” targeting) depends not only on structural plausibility but also on transparent reporting of covariate selection, goodness-of-fit/model diagnostics, and uncertainty characterization-areas where the broader literature has historically shown reporting deficiencies and a need for clearer guidance (Dartois et al., 2007).

Across the included studies, dosing accuracy is quantified using prediction-error metrics: Marsh et al. (1991) report propofol delivery performance improving from bias −18.5%/precision 25.4% to bias 0.9%/precision 20.1% after deriving a pediatric microconstant system. Accuracy is also reported via MDPE/MDAPE, including Mertens et al. (MDPE −15% to 1%; MDAPE 19%–30%), Sam et al. (2009) (MDPE 2.44%; MDAPE 21.6%), and Iirola et al. (2012), where covariates reduce MDPE from −5.9% to −3.7% and MDAPE from 33.5% to 21.7%. For dosing in obesity, the remifentanil paper (obese vs. lean) supports the conclusion that dosing regimens should be based on lean body mass (LBM). Finally, van Heusden et al. (2018) explicitly includes an optimization step to minimize the two-norm of the worst-case error, reflecting optimization in a control-theoretic sense.

Overall, these results indicate that the most practically useful advances across the reviewed models arise from (i) reducing systematic bias toward zero and (ii) tightening dispersion around the target (improved precision/MDAPE), achieved through population covariates and physiologically appropriate scaling (e.g., LBM in obesity), and-in control oriented studies-through explicit optimization of worst-case tracking error.

## 8 Conclusion

This review summarizes the development of anesthetic pharmacokinetic–pharmacodynamic (PK–PD) modeling from 1987 to 2024, with emphasis on compartmental PK structures, effect-site/link (biophase) representations of delayed drug effect, and population (nonlinear mixed-effects) modeling practices. Across the included studies, recurring themes include the use of covariates and scaling approaches to address inter-individual variability and to support prediction in clinically relevant dosing contexts. The reviewed models were compared with respect to structural assumptions, estimation approaches, reported evaluation metrics, and stated limitations. Overall, the literature indicates continued reliance on compartmental/effect-site frameworks for clinically interpretable prediction, while highlighting the importance of consistent validation and reporting to improve cross-study comparability and to support future model development and evaluation.

## 9 Possible research areas

Several obstacles exist within PK and PD modeling, including: 1) Personalization for Special Populations: Models for pediatric and obese populations require further refinements to provide accurate dosing; 2) Multi-Drug Interactions: More thorough models are needed to predict interactions in multi-drug regimens; 3) Real-Time Adaptation: Models must be capable of making real-time adaptations to reflect changing clinical situations; 4) Genetic Variability: Future models should include genetic components to enable personalized dosing; 5) Co-morbidities: The effect of co-morbidities on pharmacokinetics requires additional investigation; 6) Long-Term Predictions: Improving long-term drug prediction for a variety of patients is critical; and 7) Computational Efficiency: Models must be simplified for clinical applications. This research reviewed the development and evolution of PK-PD modeling for anesthetic care from 1987 to 2024, focusing on more complex predictive modeling methods, such as the transition from simple three-compartment models to complex multi-drug control systems. The ability of significant contributions, such as physiologically based modeling, allometric scaling, and patient-specific variables, to enhance patient safety, predictive validity, and dosage accuracy was assessed. The study also analyzed several models, discussed their advantages and disadvantages, and suggested possible research directions for creating new models of customized, closed-loop anesthetic delivery systems. Use of AI tools declaration.
