# Correction: Advances in pharmacokinetic-pharmacodynamic modeling for anesthesia, 1987–2024: a review

**DOI:** 10.3389/fphar.2026.1804067

**Published:** 2026-02-11

**Authors:** Yara Tulbah, Ibrahim Aljamaan

**Affiliations:** Biomedical Engineering Department, College of Engineering, Imam Abdulrahman Bin Faisal University, Dammam, Saudi Arabia

**Keywords:** anesthesia control, dose optimization, nonlinear mixed-effects, patient-specific covariates, pharmacodynamic prediction, pharmacokinetic modeling, population variability

The figures were presented in the incorrect order in the published PDF and HTML versions of this article. Specifically, Figures 3–5 were incorrectly placed, resulting in a mismatch between the figure numbering and their chronological sequence. The figure captions were correct. The correct figure sequence follows a chronological order based on publication periods: 1987, 1991–1999, 2000–2009, 2010–2014, 2015–2019, and 2020–2024. The correct Figures 3–5 and their captions appear below.

**FIGURE 3:**
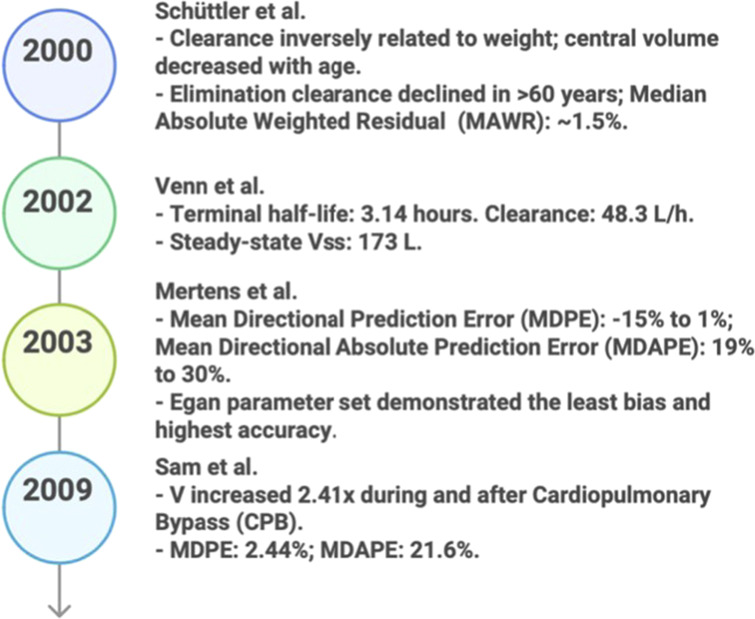
Key predicative studies results in PK/PD (2000–2009).

**FIGURE 4:**
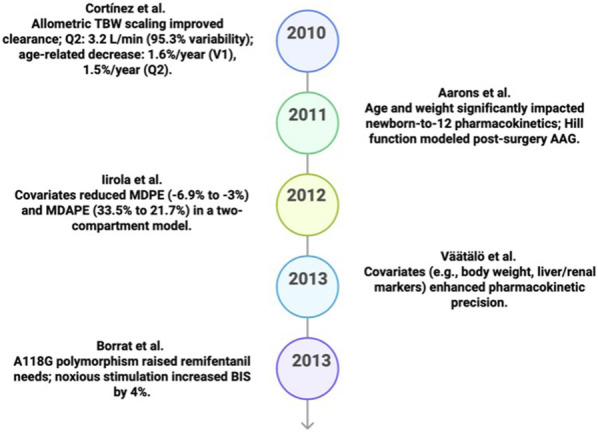
Key predicative studies results in PK/PD (2010–2014).

**FIGURE 5:**
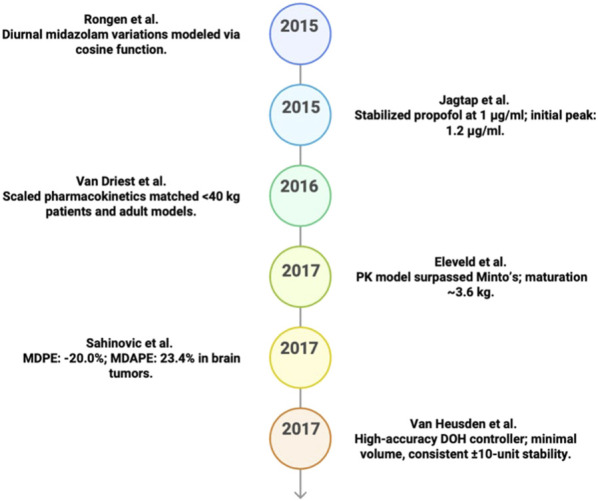
Key predicative studies results in PK/PD (2015–2019).

The original article has been updated.

